# The Obesity–Impulsivity Axis: Potential Metabolic Interventions in Chronic Psychiatric Patients

**DOI:** 10.3389/fpsyt.2017.00020

**Published:** 2017-02-13

**Authors:** Adonis Sfera, Carolina Osorio, Luzmin Acosta Inderias, Victoria Parker, Amy I. Price, Michael Cummings

**Affiliations:** ^1^Patton State Hospital, Psychiatry, Patton, CA, USA; ^2^Loma Linda University, Psychiatry, Loma Linda, CA, USA; ^3^Patton State Hospital, Medicine, Patton, CA, USA; ^4^Patton State Hospital, Dietary, Patton, CA, USA; ^5^Oxford University, Evidence Based Medicine, Oxford, UK

**Keywords:** monoamines, acetylcholine, short-chain fatty acids, impulsivity, microbiome

## Abstract

Pathological impulsivity is encountered in a broad range of psychiatric conditions and is thought to be a risk factor for aggression directed against oneself or others. Recently, a strong association was found between impulsivity and obesity which may explain the high prevalence of metabolic disorders in individuals with mental illness even in the absence of exposure to psychotropic drugs. As the overlapping neurobiology of impulsivity and obesity is being unraveled, the question asked louder and louder is whether they should be treated concomitantly. The treatment of obesity and metabolic dysregulations in chronic psychiatric patients is currently underutilized and often initiated late, making correction more difficult to achieve. Addressing obesity and metabolic dysfunction in a preventive manner may not only lower morbidity and mortality but also the excessive impulsivity, decreasing the risk for aggression. In this review, we take a look beyond psychopharmacological interventions and discuss dietary and physical therapy approaches.

## Introduction

Although the prevalence of obesity and metabolic syndrome has been increasing worldwide over the past decades, it is significantly higher in mentally ill patients regardless of exposure to psychotropic drugs (PTDs) (1–9). In this regard, it was hypothesized by others that the parallel growth of psychiatric and metabolic disorders may indicate a shared pathoetiology (10–13).

Pathological impulsivity encompasses a heterogenous group of psychiatric disorders characterized by inability to resist impulses for engaging in behaviors harmful to self or others. It is encountered in numerous psychiatric conditions, ranging from intermittent explosive disorder, pathological gambling, kleptomania, trichotillomania, and pyromania on the one hand, to schizophrenia, mania, attention-deficit hyperactivity disorder (ADHD), antisocial personality disorder, and drug addictions on the other. The psychopharmacological treatment of these disorders is dependent on the primary pathology and may include almost all classes of PTDs: serotonin reuptake inhibitors, stimulants, antipsychotics, mood stabilizers, and in some instances opioid antagonists.

This observation is in line with the epidemiological studies, linking pathological impulsivity with weight gain and dysmetabolism (14–19). There is a growing body of evidence that includes studies in young individuals, indicating that obese/overweight adolescents are more likely to engage in risky behaviors (20). Along the same lines, nutritional studies reveal that perinatal exposure to high fat diets is more likely to bring about impulsive offspring (21, 22). Other research in nutrition connected a high trans-fat diet with aggression, while sociological studies pointed to a correlation between antisocial behavior and obesity (23, 24). Neuroimaging studies shed additional light on the impulsivity–obesity connection as functional magnetic resonance imaging and diffusion tensor imaging studies documented lower perfusion in the orbitofrontal, medial/ventrolateral prefrontal cortex, and middle/superior frontal gyri in impulsive and obese individuals (25–28). In addition, this body of evidence includes novel endocrinology studies demonstrating dysregulation of metabolic hormones ghrelin, leptin, and adiponectin in conditions associated with pathological impulsivity, including ADHD, aggression, and antisocial personality disorder (29–34). On the other hand, clozapine, an established anti-impulsivity drug, was found to directly alter the expression of leptin and adiponectin genes (35–37). Lithium, another anti-impulsivity drug, was found beneficial in correcting the glycemic parameters in diabetes mellitus type 2 (T2DM) by inhibiting glycogen synthase kinase-3 (GSK-3) a leptin-lowering enzyme (38–40). As mentioned earlier, lower leptin levels were associated with excessive impulsivity and dyslipidemias (41).

Multiple psychiatric studies over the past decades established that acetylcholine (ACh) and monoamines (MAs) including norepinephrine, dopamine, histamine, and serotonin are involved in a wide range of psychiatric disorders, including those marked by pathological impulsivity (14, 42–44). As these neurotransmitters were demonstrated to also modulate the hypothalamic feeding centers, it should not come as a surprise that impulsivity and obesity are intertwined (45, 46). Indeed, most antiobesity drugs present with agonistic action at the receptors in which many PTDs antagonize, suggesting the possibility of a psychometabolic continuum (46).

The development of PTDs with fewer metabolic adverse effects currently represents a major unmet need in psychiatry. As obesity and metabolic dysfunction in psychiatric patients trigger higher morbidity and mortality rates than in the general population, there is a heightened urgency for their prevention and early correction. Therefore, until this need is met, optimal utilization of available pharmacological and non-pharmacological tools is crucial for the overall well-being of these patients.

At present, the treatment of metabolic disorders in psychiatric patients is not only underutilized but also frequently delayed, rendering correction more difficult to achieve (6, 7). Indeed, after the diagnosis of obesity or metabolic syndrome is established, there are few effective interventions for reversing these conditions. In general, these interventions revolve around replacing an orexigenic with a less orexigenic drug, at the risk of clinical destabilization, or lifestyle and behavioral changes that have proved difficult to implement in this population (47, 48). Therefore, clinicians should adopt preemptive approaches, striving to avoid or delay the onset of obesity, and the metabolic syndrome instead of correcting them *post hoc* (7).

More studies are needed to assess the efficacy of preventive metabolic interventions, including the utilization of available antiobesity drugs in psychiatric disorders and PTD-induced obesity. There are even fewer studies on alternative modalities, including nutrition, physical therapy, and parasympathetic stimulation *via* cholinesterase inhibitors or transcutaneous auricular vagal nerve stimulation (taVNS).

In this article, we will review some of these modalities after a brief discussion on antiobesity drugs and their action in the hypothalamic feeding centers.

## Arcuate (ARC) Nucleus: Where Psychopharmacology and Antiobesity Pharmacopeias Collide

Hunger, satiety, and energy homeostasis are controlled by the neuronal networks in the mediobasal hypothalamus and some brainstem areas. The ARC nucleus of the hypothalamus contains the first order neurons that balance hunger and satiety in order to maintain a stable body weight (49). This balancing act requires a constant cross-talk between the ARC nucleus and the peripheral organs, which takes place *via* metabolic hormones, neural input, and neurotransmitters, including MA and ACh (50).

**Figure 1:**
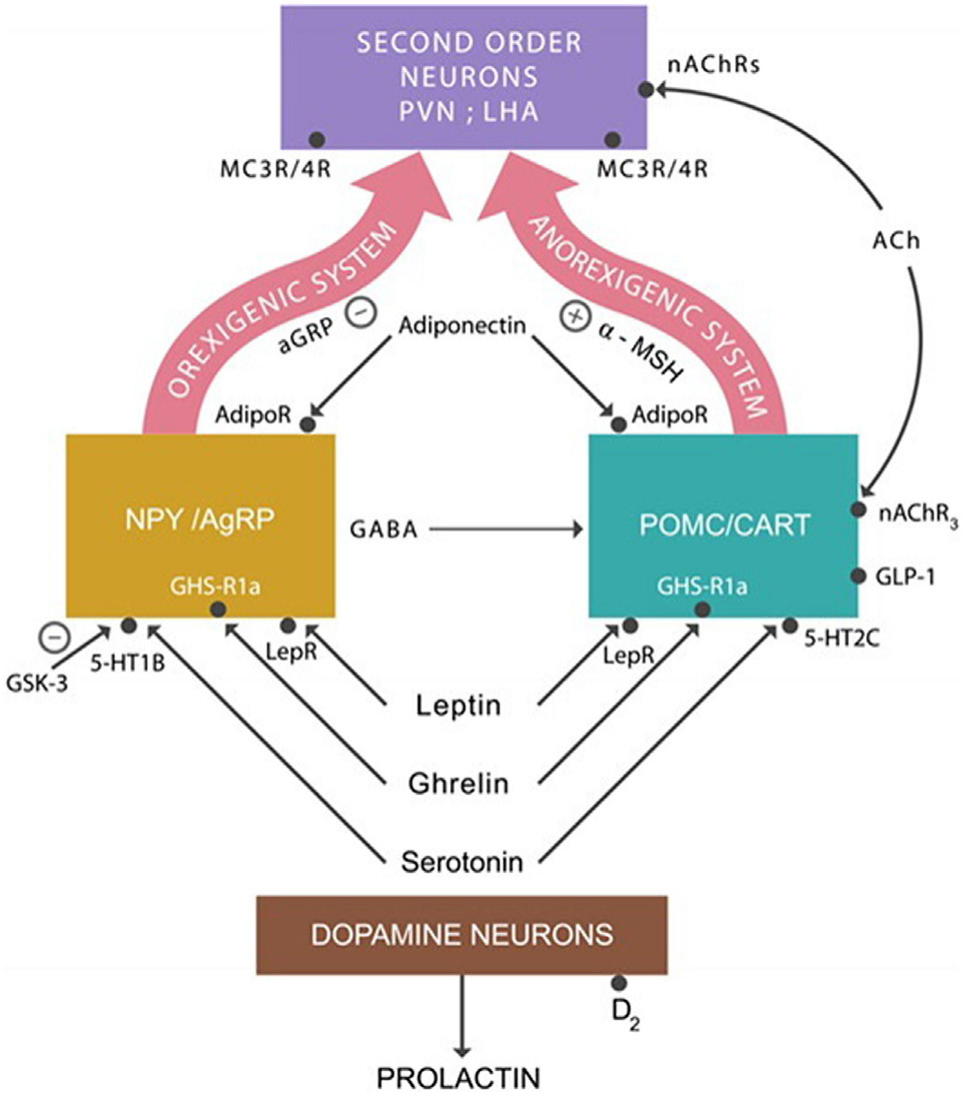
**The anorexigenic system: alpha-melanocyte stimulating hormone (alpha-MSH) activates MC3/4 receptors expressed on second order neurons, inhibiting appetite**. The orexigenic system: agouti-related protein (AgRP) is a competitor of alpha-MSH at MC3/4 receptors, augmenting appetite. Adiponectin, leptin, ghrelin, and serotonin activate the anorexigenic system. The action of these hormones on the orexigenic system results in the release of the inhibitory neurotransmitter GABA, which applies the brake on the anorexigenic system, increasing appetite. Abbreviations: AdipoR, adiponectin receptors; nAChRs, nicotinic cholinergic receptors; LepRs, leptin receptors; GHS-R1a, ghrelin receptors; serotonin 5-HT2C and 5-HT1A receptors.

The anorexigenic system that lowers appetite is composed of proopiomelanocortin (POMC) and cocaine- and amphetamine-regulated transcript (CART) neurons. They produce the alpha-melanocyte stimulating hormone (alpha-MSH) that binds to the melanocortin 3 and 4 receptors (MC3Rs and MC4Rs) expressed by the second order neurons located in the periventricular nucleus and the lateral hypothalamic area (LHA) (51) (Figure 1).

The orexigenic system, which increases the appetite, consists of neuropeptide Y (NPY) and agouti-related protein (AgRP). They produce NPY, GABA, and the AgRP. AgRP is an antagonist of alpha-MSH at MC3Rs and MC4Rs prevents their activation, whereas GABA inhibits the POMC/CART neurons. Both of these actions result in appetite augmentation.

In addition to these neuronal groups, ARC also contains dopamine neurons which project to the anterior pituitary gland where they inhibit prolactin secretion (Figure 1). Blocking dopamine-2 (D-2) receptors by PTDs often results in hyperprolactinemia, a common adverse effect of high potency antipsychotic drugs (52).

Over the past few years, new drugs indicated for the long-term treatment of obesity obtained FDA approval. Interestingly, two of them are combinations of PTDs utilized in the treatment of psychiatric disorders. For example, phentermine–topiramate extended release (Qsymia) and naltrexone–bupropion extended release (Contrave) have been prescribed individually in affective disorders and drug addictions. These drugs may have a place in the preventive treatment of PTD-induced obesity as they may be prescribed “off label” in select patients needing more adequate mood stabilization. In this regard, four large randomized, double-blind, placebo controlled trials support the preventive use of topiramate alone for PTD-induced weight gain (53). Concomitant utilization of topiramate with orexigenic PTDs may constitute a preemptive intervention to avoid or delay the onset of obesity and metabolic dysregulation (Table 1). About 10% of patients may experience mild to moderate cognitive adverse effects early on topiramate treatment with verbal fluency being more affected compared to other antiepileptic drugs.

**Table 1:** **Antiobesity and anti-diabetes mellitus type 2 (T2DM) drugs for potential use in psychiatry**.

Combination drugs	FDA approved for
Phentermine/topiramate extended release (QSYMIA)	Obesity long term
Bupropion/naltrexone extended release (CONTRAVE)	Obesity long term
**Glucagon-like peptide-1 agonists**	
Liraglutide (Victoza)	T2DM + obesity long term
Exenatide (Byetta)	T2DM (possible antiobesity action)
Albiglutide (Tanzeum)	T2DM (possible antiobesity action)
**Monoamine agonists**	
Phentermine, diethylpropion, benzphetamine	Obesity (short term)
Bromocriptine (Cycloset)	T2DM (possible antiobesity action)
Amantadine (Symmetrel)	Influenza A, Parkinson’s disease (possible antiobesity action)
Locaserin	Antiobesity long term
**Cholinergic agonists and enhancers**	
Sofinicline	(Developmental stage-antiobesity)
Donepezil, rivastigmine, galantamine	Alzheimer’s diseases (possible antiobesity and anti T2DM action)
**Histamine agonists**	
Betahistine	Meniere’s disease (possible antiobesity and anti T2DM action)
Melanin-concentrating hormone antagonists	(Developmental stage-antiobesity)

*In parenthesis: non-FDA approved*.

## Metformin

Metformin is a drug widely prescribed in psychiatric patients diagnosed with T2DM, but rarely in a preventive manner, even though several studies found this drug to be efficient in preventing both obesity and T2DM (54–56). Since metformin is known for its relatively benign adverse effects, it could be prescribed from the treatment onset along with the orexigenic PTDs.

Metformin was demonstrated to result in 2.93–5 kg weight loss after 6 months of treatment even when prescribed after the onset of metabolic dysregulation (55). More studies are needed to assess the benefits of preventive metformin use alone or in combination with other antiobesity agents.

## Glucagon-Like Peptide-1 (GLP-1)

Recently, there has been a strong interest in the GLP-1 agonists, including liraglutide (Victoza), exenatide (Byetta), and albiglutide (Tanzeum). These agents have been shown effective in halting weight gain in diabetic patients and their antiobesity action is most likely mediated *via* GLP-1 receptors expressed on POMC/CART neurons (57) (Figure 1).

In addition, novel studies demonstrate that GLP-1 inhibition may mediate clozapine-induced weight gain and metabolic dysregulation, suggesting that GLP-1 agonists could reverse both (58). To the best of our knowledge, currently, there is one ongoing study aiming at exploring liraglutide’s effects on glucose tolerance in patients on clozapine or olanzapine treatment (59). The once-a-week drug exenatide did not promote weight loss in this population (60). As liraglutide was demonstrated to be more effective than the GLP-1 mimetics, a clinical trial should be initiated to assess the efficacy of this drug for PTD-induced obesity either alone or in combination with another compound, such as locaserin (61). The once-a-week albiglutide was also not tested for weight loss in PTD-induced obesity. A large study found this drug effective, especially as an adjunct to metformin, suggesting that it could be helpful for psychiatric patients with inadequate glycemic control on metformin alone (62). In addition, since liraglutide has both T2DM and weight loss indication, it could be prescribed more often in overweight/obese psychiatric patients, especially when other agents cannot achieve adequate glycemic stabilization. Interestingly, novel studies demonstrate that GLP-1 agonists may have PTD-synergistic actions on impulsivity, suggesting that liraglutide-treated psychiatric patients may be managed with lower PTDs doses (63–67). Furthermore, since GLP-1 signaling was suggested as a common pathophysiological mechanism in both Alzheimer’s disease and T2DM, GLP-1 agonists may prevent cognitive decline (68).

## Locaserin

The novel antiobesity drug, locaserin, is an agonist at serotonin 5-HT2C receptors that are antagonized by some PTDs, inducing the adverse effect of weight gain. Interestingly, preclinical studies demonstrated the efficacy 5-HT2C agonists for the treatment of excessive impulsivity (46, 69–71).

Different studies point to a connection between serotonin signaling and the metabolic hormones—ghrelin, leptin, and adiponectin—which were also linked to impulsivity (29–34, 41). For example, studies in rodents show that leptin modulates the biosynthesis and release of serotonin by the raphe nuclei (72–74). Other studies report that leptin receptors (LepRs) act synergistically with 5-HT2C expressed on POMC/CART neurons and with 5-HT1B receptors on NPY/AgRP neurons (75) (Figure 1). Clozapine, a drug with known anti-impulsivity actions and orexigenic adverse effects, is an antagonist at both 5-HT2C and 5-HT1B receptors. In addition, as mentioned earlier, clozapine alters the expression of leptin and adiponectin genes, suggesting direct effects on both impulsivity and weight gain.

To the best of our knowledge, locaserin has not been tested in PTD-induced weight gain, but its receptor profile suggests it may be beneficial as an adjunct in clozapine-, olanzapine-, or mirtazapine-treated patients.

A body of literature links the reduced serotonergic 5-HT1B receptor activity with impulsivity and drug addictions (76–79). In the hypothalamus, the 5-HT1B receptors are expressed by the NPY/AgRP neurons and their activation inhibits the release of GABA, thus disinhibiting the anorexigenic system, inducing appetite suppression (Figure 1). The 5-HT1B receptors were demonstrated to act synergistically in appetite suppression with the 5HT2C at POMC/CART receptors (75). At present, there are no concomitant agonists at 5-HT2C and 5-HT1B receptors, but such drugs may be expected to present with superior antiobesity and anti-impulsivity actions. As discussed above, lithium, a major anti-impulsivity drug, inhibits GSK-3, a 5-HT1B-blocking enzyme. Over-expressed GSK-3 was associated with excessive impulsivity in multiple psychiatric syndromes, including mania, suicide, and schizophrenia (80–84). In addition, GSK-3 upregulation was demonstrated in obesity and insulin resistance (38, 39, 85, 86). Since lithium was documented to possess anti-diabetic properties, GSK-3 inhibitors are currently T2DM targets (40). Indeed, three decades ago, it was noted that lithium acted synergistically with some antidiabetic drugs; however, the mechanism was unknown at that time (87–89) (Table 1).

## Dopaminergic Drugs in Obesity–Impulsivity Axis

Some of the FDA approved antiobesity agents are agonists at dopamine D-2 receptors that are antagonized by all clinically utilized antipsychotic drugs (90). The antiobesity D-2 agonists include phentermine, diethylpropion, benzphetamine, and bupropion (as part of the combination drug Contrave). In addition, the D-2 stimulants bromocriptine and amantadine have been associated with weight loss (91, 92). For example, amantadine has demonstrated efficacy in overweight/obese patients treated with olanzapine (91). Bromocriptine under the market name Cycloset recently obtained FDA indication for the treatment of T2DM. This drug has been underutilized in PTD-associated glycemic dysfunction, although it may be beneficial for diabetic patients requiring treatment with risperidone, paliperidone, or some antidepressants that are often associated with hyperprolactinemia.

**Figure 2:**
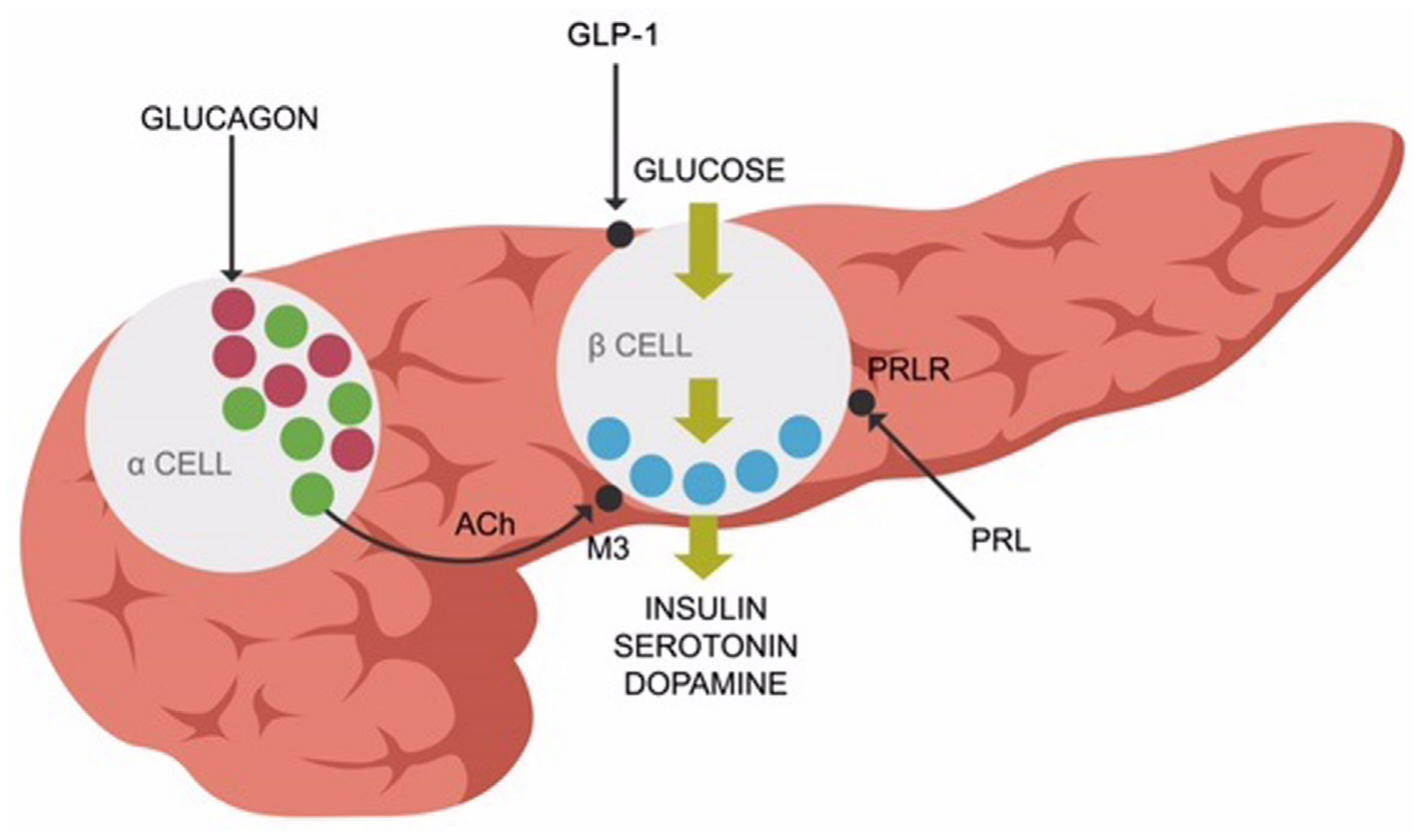
**Prolactin receptors (PRLR), muscarinic-3 cholinergic, and glucagon-like peptide-1 (GLP-1) receptors are expressed on pancreatic beta-cells, facilitating insulin release along with serotonin and dopamine**.

Recent studies revealed that elevated prolactin induces pancreatic beta cells to secrete insulin. Chronic hyperprolactinemia induces hyperinsulinemia, eventually resulting in beta cells exhaustion and T2DM (Figure 2). Aside from the anterior pituitary, prolactin is also released by the adipose tissue as a pro-inflammatory cytokine demonstrated to augment the metabolic inflammation associated with obesity (93, 94). This is one of the primary reasons clinicians should address hyperprolactinemia promptly in PTD-treated patients, after prolactinomas have been ruled out (94). Novel studies do not support the older concept that bromocriptine or other D-2 stimulants invariably precipitate psychosis. Persistently elevated prolactin was demonstrated by large studies to increase the all-cause mortality, emphasizing the need for treating this condition promptly (95, 96). In addition, other studies demonstrated the benefit of D-2 stimulation in patients with negative symptoms of schizophrenia (97, 98). Furthermore, some researchers hypothesized that hyperprolactinemia may exacerbate both schizophrenia and T2DM (99).

Appetite contains a large hedonic component mediated by the central reward system. A striatal hypodopaminergic state is believed to trigger overnutrition, which in turn augments dopamine transmission. According to this model, D-2 stimulants correct the hypodopaminergia, lowering the overnutrition reward (100–102). Indeed, reduced D-2 receptor density was demonstrated in obese individuals, implying impaired dopaminergic signaling in this metabolic disorder (103). Psychiatric patients treated with antipsychotic drugs (D-2 blockers) may present with an enduring iatrogenic hypodopaminergic state, which in turn triggers overnutrition to augment dopamine levels.

Amantadine, bromocriptine, or phentermine–topiramate extended release should be encouraged in psychiatric patients, especially when presenting with extrapyramidal symptoms or elevated prolactin.

Lisdexamfetamine, a dopaminergic pro-drug metabolized to dexamphetamine, was approved by the FDA for ADHD; however, its appetite suppressant activity and low abuse potential may render it useful in PTD-induced obesity. Recently, the effectiveness of this compound was demonstrated in binge eating (104). In addition, its stimulant action was associated with significant improvements in negative symptoms of schizophrenia without worsening the positive symptoms (105).

## Acetylcholine

A novel line of research demonstrates that ACh signaling and metabolic pathways intersect, both in the pancreas and the hypothalamus (106, 107). Centrally, nicotine activates the nicotinic cholinergic receptors (nAChRs) expressed on POMC/CART neurons, turning the anorexigenic system “on” and lowering appetite (Figure 1). This was demonstrated to take place in smokers, while following smoking cessation, the lack of anorexigenic activation heightens the appetite, lading to weight gain. In addition, as nAChRs are also expressed on the second order neurons in the LHA and the perifornical organ. Smoking discontinuation affects these areas also, inducing weight gain (108).

Peripherally, ACh enables insulin release (in response to glucose) from the pancreatic beta cells *via* muscarinic-3 (M-3) receptors. Pancreatic alpha cells secrete ACh, facilitating the release of insulin from the beta cells (109, 110) (Figure 2).

As several PTDs are known to block the M-3 receptors, they may induce glycemic dysregulation (111). Interestingly, insulin is synthesized and released from the beta cells along with serotonin and dopamine molecules, demonstrating once more the interconnectedness between energy metabolism and ACh/MAs. This finding is in line with the epidemiological studies associating smoking cessation with increased risk for T2DM for up to 12 years (112).

In light of these data, it should not come as a surprise that obesity and T2DM have been associated with the over-expression of Ach-degrading enzymes—butyrylcholinesterase (BChE) and acetylcholinesterase (AChE)—which tone down cholinergic signaling. Indeed, cholinesterase inhibitors—donepezil, rivastigmine, and galantamine—were found beneficial in metabolic syndrome by some researchers (113–115). In addition, these drugs are currently utilized in patients with Alzheimer’s disease for the treatment of aggression, suggesting a psychometabolic role (116). Preventive use of these agents in psychiatric patients could preempt the development of metabolic dysfunction and obesity. Rivastigmine that inhibits both AChE and BChE was found to be a more effective anti-impulsivity agent in dementias (117). Interestingly, BChE was demonstrated to hydrolyze both ACh and ghrelin, thus comprising an antiobesity/impulsivity target (114, 118, 119). Moreover, LepRs were demonstrated to act synergistically with both serotonin and ACh receptors on POMC/CART neurons, indicating a close coordination between the metabolic hormones and these neurotransmitters (75). Furthermore, the existence of a leptin–dopamine axis was implied by preclinical studies which found that mesolimbic leptin signaling modulates the CNS reward system (120).

Melatonin and serotonin 5-HT2C receptors were demonstrated to be coexpressed in various areas of the CNS, possibly including POMC/CART neurons, explaining the frequently discussed antiobesity effect of melatonin (121).

The impaired expression of CHRNA gene, coding for the nicotinic acetylcholine receptors, has been associated with disorders marked by excessive impulsivity, including ADHD, drug addictions, and antisocial personality disorder (43, 122, 123). Sofinicline, a novel nicotinic agonist believed to augment CHRNA expression, is currently being tested for ADHD but appears promising in metabolic syndrome, T2DM, and eating disorders (124–126). The metabolic action of sofinicline is probably due to ghrelin modulation of POMC/CART neurons *via* nicotinic receptors coexpressed with 5-HT2C (33, 127). This recently revealed receptor–receptor interaction may lead to the development of novel compounds for metabolic syndrome and impulsivity (33, 128–130).

Clinicians should be encouraged to prescribe cholinesterase inhibitors in a preventive manner in psychiatric patients susceptible to PTD-induced obesity and dysmetabolism, especially in the presence of schizophrenia or dementia-related cognitive impairments. In this respect, rivastigmine patch represents a low risk intervention with several advantages over oral medications. Moreover, since impaired cholinergic signaling was involved in both metabolic and impulsivity disorders, clinicians should avoid the use of anticholinergic drugs even in younger patients and consider replacing them with amantadine for the extrapyramidal adverse effects of antipsychotic drugs and with melatonin or ramelteon for insomnia. Furthermore, cholinergic signaling may be enhanced *via* taVNS, which represents another low risk intervention for improving insulin sensitivity (131). Interestingly, taVNS has documented efficacy in depression, which may represent an additional benefit for psychiatric patients (132).

## Histamine

The relationship between histamine and metabolism was first described in the 1950s, yet the development of histaminergic treatments for metabolic disorders and obesity is still in its infancy (133). The blockade of histamine-1 (H-1) receptors by PTDs was associated with both obesity and T2DM, suggesting the involvement of histaminergic pathways in energy homeostasis (131, 134–136). Briefly, H-1 and H-2 receptors’ blockade induces sedation, while antagonism at H-3 receptors results in wakefulness, as H-3 receptors are primarily autoreceptors.

Some clinical and preclinical studies demonstrated that betahistine, a centrally acting H-1 agonist and partial H-3 antagonist, was effective as an antiobesity drug, especially in younger women (137). Studies with olanzapine-induced obesity in rodents also demonstrated weight loss after exposure to betahistine (138). However, as other studies failed to establish a direct link between histamine and body weight, an indirect action is now suspected *via* other neurotransmitter systems or metabolic hormones, including thyrotropin-releasing hormone, growth hormone, and leptin (139–141). Indeed, novel human studies report that H-1 receptor antagonists inhibit alpha 7nAChRs which turn “off” the POMC/CART anorexigenic system (139). As alpha 7nAChRs are involved in cognition, histamine-blocking agents (including some PTDs) may represent both metabolic and cognitive risk factors.

Aside from these indirect actions on feeding, histamine was recently demonstrated to directly control the orexigenic melanin-concentrating hormone (MCH) (142). Indeed, the MCH receptor antagonists are currently thought of as promising antiobesity agents (143). As MCH neurons express H-3, but not H-1 or H-2 receptors, it is likely that histamine inhibits MCH secretion *via* H-3 receptors. Several studies indicate that histamine also exerts H-3-mediated anti-diabetic properties (144–146) (Table 1).

Furthermore, human microglial cells were documented to express H-3 receptors that are responsible for their activation (147). As numerous neuropsychiatric conditions were linked to microglial activation, H-3 receptors’ antagonists are currently evaluated for their efficacy in schizophrenia and Alzheimer’s disease (147, 148). Moreover, novel studies suggest that H-3 blockade may be associated with decreased impulsivity, especially in dementia, Parkinson’s disease, ADHD, and drug addiction (149–152). As histamine is known to be modulated by leptin and reduced leptin was associated with impulsivity, these finding are in line with the research connecting histamine with obesity–impulsivity axis (30–32).

## The Brain–Gut Axis: May the Microbes be with You

Dietary interventions have been extremely underutilized in psychiatric patients despite accumulating evidence for their benefits.

The Alternate Healthy Eating Index 2010 (AHEI-2010) is a measure of diet quality. A high AHEI score was documented to lower the risk of chronic disease, including T2DM and cardiovascular disease and to reverse the metabolic syndrome (153–155). High AHEI score diets have demonstrated benefits in the affective and cognitive disorders, but they were not studied in schizophrenia or PTD-induced obesity (156, 157). These diets are thought to exert their beneficial effects by stabilizing the gut microbiome.

The human microbiome consists of over 100 trillion bacteria, fungi, and protozoa which inhabit the gastrointestinal (GI) tract, living in symbiosis with the host cells. Several lines of evidence point to the potential role of gut microbiota in modulating not only the host energy metabolism but also information processing and behavior (158, 159). Human and animal studies demonstrated that obesity was associated with GI “overpopulation” with phylum Firmicutes and concomitant decreases in phylum Bacteriodetes (160, 161). Interestingly, administration of an antibiotic along with olanzapine minimized the amount of the weight gain in rodents, a finding that may be in line with the antibiotic minocycline reducing psychotic symptoms in patients with schizophrenia. Minocycline is a tetracycline that was demonstrated to not only modulate the dopaminergic and glutamatergic CNS signaling but also to restore the physiologic Firmicutes/Bacteroidetes ratio in the gut with positive effect on hypertension (162).

These studies suggest that that gut microbiota may be involved not only in the etiology of obesity but also in schizophrenia, perhaps explaining the predisposition to metabolic disorders encountered in psychiatric patients (163, 164). In addition, preclinical studies show that transplantation of GI tract microorganisms from the obese into the lean mice was followed by weight gain in the later. Interestingly, fecal analysis of olanzapine treated rodents, demonstrated increases in Firmicutes and decreases in Bacteriodetes phyla, a pattern identical to the one found in obese humans (165, 166).

Other novel studies linked low dietary fiber with the dysregulation of Firmicutes/Bacteriodetes ratio. The colonic microbiota is known to induce fermentation of dietary fiber, resulting in the production of beneficial short-chain fatty acids (SCFAs), including butyrate and propionate. The SCFAs deficiency is believed to represent a risk factor in the pathogenesis of obesity (167). In this respect, butyrate was found protective against insulin resistance and inflammation, while propionate was demonstrated to lower cholesterol synthesis (168). Moreover, SCFAs were shown to augment the production of leptin which, as discussed above, was found to be decreased in disorders marked by impulsivity, thus linking SCFAs with psychiatric conditions (169).

Short-chain fatty acids were demonstrated not only to reach the bloodstream but also to cross the blood–brain barrier (BBB) and alter the hypothalamic leptin and adiponectin gene expression (170, 171). Activation of adiponectin receptors, expressed on both POMC/CART and NPY/AgRP are known to potentiate the function of leptin, lowering impulsivity (172, 173). Interestingly, many patients with schizophrenia treated with second-generation antipsychotic drugs were found to have lower plasma adiponectin and leptin levels (174, 175). Studies in metabolism indicate that hypoadiponectinemia lowers the skeletal muscle uptake of postprandial glucose (insulin resistance), eventually leading to T2DM (176, 177). Moreover, supplementation with dietary fiber was demonstrated to increase adiponectin levels by up to 115%, suggesting that SCFAs play a key role in adiponectin biosynthesis (178).

Western diets in general are known for lacking adequate amounts of dietary fiber. For example, typical Western adults were shown to consume 5–10 g of fiber daily, as opposed to the 35 or 50 g which is considered optimal (179). Patients with schizophrenia were demonstrated to consume an even lower amount of dietary fiber compared to the overall Western population, perhaps explaining the low adiponectin levels documented in this group (180). Mediterranean diets and variants aside from their demonstrated anti-inflammatory actions were found to facilitate SCFAs biosynthesis (181) (Table 2).

**Table 2:** **Dietary interventions in metabolic syndrome**.

Dietary intervention	Reference
Adherence to Alternate Healthy Eating Index-2010	(153, 154)
Anti-inflammatory diets	(154, 157)
Adiponectin-increasing diets	(178, 182)
High dietary fiber modifications	(183, 184)
Dietary polyphenols	(185, 186)

Dietary polyphenols were documented to help maintain the adequate gut microbial balance (187). In addition, polyphenols were shown to be protective against T2DM and possibly psychiatric disorders manifested by impulsivity (182–186). To the best of our knowledge, at present, there are no studies on polyphenol/high fiber diets in psychiatric or PTD-treated patients. We suggest a trial with a modified Mediterranean diet, incorporating high dietary fiber, polyphenols, and SCFA. The efficacy of this approach can be monitored not only *via* BMI and the glycemic parameters but also by plasma adiponectin and leptin levels.

## Skeletal Muscle and the “Exercise Factors”

In addition to enabling the human body mobility, the over 600 skeletal muscles comprise a complex endocrine organ, known for the secretion of a growing number of “myokines” which exert both local and distal effects. “Exercise factors” are a subgroup of myokines released into the circulation during the exercise (188, 189). Among them, the peroxisome proliferator-activated receptor-γ coactivator 1alpha (PGC-1alpha) is the most studied as it was shown to increase both the skeletal muscle glucose uptake and the oxidation of fatty acids (190). A sedentary lifestyle with lowered expression of PGC-1alpha is associated with weight gain, inflammation, and decreased insulin secretion, pathological changes that eventually culminate in T2DM and obesity (191) (Table 3).

**Table 3:** **Specific therapeutic modalities described in metabolic syndrome**.

Therapeutic modalities	Reference
Transcutaneous vagus nerve stimulation	(132, 192)
Isometric exercises	(193)
Swiss ball exercises	(194)
Whole body vibration exercises	(195)

Novel studies demonstrate the positive impact of physical exercise in disorders marked by excessive impulsivity, including ADHD, affective disorders, schizophrenia, drug addictions, and Alzheimer’s disease (196–199).

The sequence of events that may lead to T2DM is believed to be initiated by decreased uptake of glucose into the skeletal muscle or insulin resistance, followed by hyperinsulinemia which in time exhausts the beta cells, leading to insulin deficiency and T2DM. The skeletal muscle (assisted by insulin) uptakes about 80% circulating glucose immediately after meals, rapidly clearing the postprandial hyperglycemia (200). Impaired glucose uptake, elevated blood glucose levels, and hyperinsulinemia comprise act one of the T2DM drama. From this point on, it may take decades before the pancreatic beta cells become insufficient and unable to secrete insulin that triggers act two: insulin deficiency and frank T2DM. As insulin is cosecreted with serotonin and dopamine, these MAs also become deficient, contributing to circulatory and cardiovascular pathology which frequently accompany T2DM (Figure 2).

Tryptophan is an essential amino acid and the sole precursor of the human body serotonin. The CNS manufactures its own serotonin; however, exogenous tryptophan must be supplied to the brain, a process facilitated by insulin. Deficiency of this hormone manifested by lower brain tryptophan was documented in patients with T2DM, possibly explaining the higher prevalence of depression in this metabolic disorder (201). In addition, tryptophan depletion studies in humans and animals demonstrated that low plasma tryptophan levels were associated with aggression (202–204).

**Figure 3:**
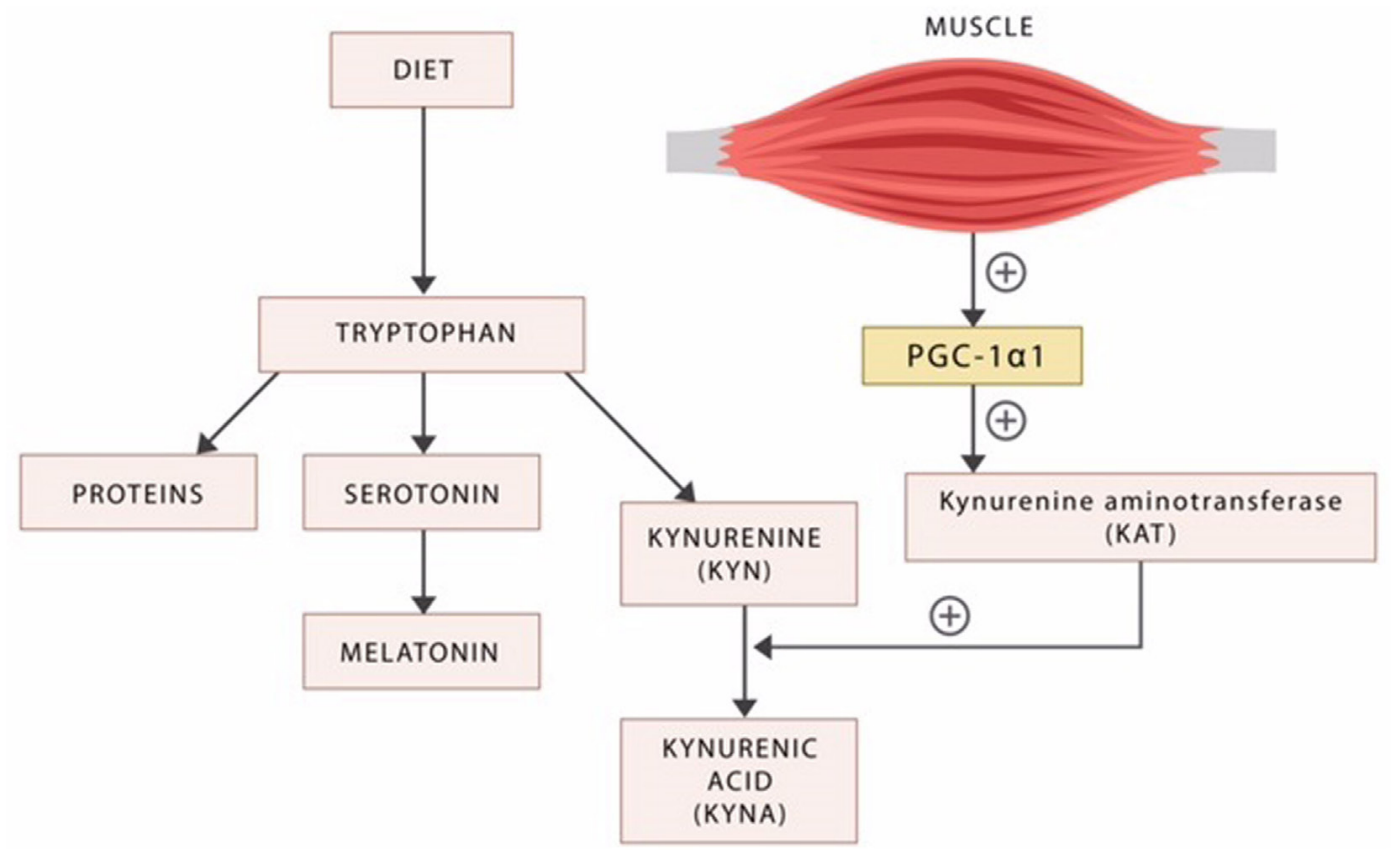
**Dietary tryptophan is the precursor of proteins, serotonin, and the kynurenines**. The exercise factor peroxisome proliferator-activated receptor-γ coactivator 1alpha (PGC-1alpha) released by the active skeletal muscle induces kynurenine aminotransferases (KAT), the enzyme responsible for the biosynthesis of the neuroprotective KYNA. In the absence of exercise, the neurotoxic KYN crosses the blood–brain barrier inducing psychopathology.

Aside from serotonin, tryptophan is catabolized into several other neuroactive compounds, including the neurotoxic kynurenine (KYN) and the neuroprotective kynurenic acid (KYNA) (Figure 3).

Kynurenine was demonstrated to cross the BBB and was implicated in the pathophysiology of disorders marked by impulsivity, including schizophrenia, ADHD, cognitive disorders, drug addictions, and mania (205). KYN is an antagonist at both the *N*-methyl-d-aspartate (NMDA) and alpha-7 nACh receptors, suggesting a possible pathoetiological mechanism of excessive impulsivity (206). This mechanism may be similar in nature to the antagonism at alpha-7 nACh receptors in POMC/CART neurons associated with weight gain and the T2DM risk (as referenced above in the discussion concerning smoking cessation).

It was recently demonstrated that the enzyme kynurenine aminotransferase (KAT) catalyzes the transformation of KYN into KYNA, in a reaction facilitated by the exercise factor PGC-1alpha (207) (Figure 3). As KYNA is neuroprotective, this molecular mechanism may provide an explanation for the known benefits of physical exercise in psychiatric disorders (208, 209) (Figure 3).

While the beneficial effect of exercise and physical activity are well established, specific recommendations for the metabolic dysregulation and regimen details are beginning to emerge (Table 2).

## Conclusion: When the Seven Lean Cows Ate up the Seven Fat Cows

The energy–behavior link was intuited by the previous generations and currently we are merely rediscovering an old truth: the brain and energy are tightly interconnected. Indeed, the energy–hungry brain utilizes over 20% of the total body energy budget for the functioning of excitatory and inhibitory synapses believed to be dysregulated in impulsivity-connected psychopathology (210).

The metabolic hormones—leptin, adiponectin, and ghrelin—communicate the peripheral energy status to the hypothalamic feeding centers *via* receptors coexpressed with the major behavioral modulators, the MAs and ACh. As these receptors are blocked by the PTDs, they may become insensitive (for example, leptin resistance), inducing hypermetric hormonal responses with resultant obesity and metabolic dysregulation.

While PTDs may correct synaptic transmission, they alter the psychometabolic continuum, inducing weight gain in individuals already predisposed to it. Here, we suggest halting the development of metabolic dysregulation and obesity by initiating preventive treatment concurrently with the PTDs. Indeed, the management of impulsive aggression in psychiatric disorders may be incomplete without addressing the metabolic component and restoring the homeostasis of the psychometabolic continuum.

Addressing psychopathology and metabolic dysfunction simultaneously may result in superior outcomes compared to an intervention that addresses these conditions in a singular way. In addition, correcting energy homeostasis may lower the overall morbidity, mortality, and institutional cost in psychiatric population. A model can be inferred from clinical practice where it was found that correcting hypertension led to better outcomes in coronary artery disease, stroke, and renal failure compared to their *post hoc* treatment. After all, as prevention is preferable to treatment, what would be a valid reason for not addressing metabolic dysregulation in psychiatric patients in a preemptive manner?

## Author Contributions

All the authors have contributed equally to this work.

## Conflict of Interest Statement

The authors declare that the research was conducted in the absence of any commercial or financial relationships that could be construed as a potential conflict of interest. The reviewer (RG) declared a past co-authorship with the authors to the handling editor and the reviewer (NS) declared her shared affiliation with the handling editor, who ensured that the process met the standards of a fair and objective review.
